# Triplet sensitization enables bidirectional isomerization of diazocine with 130 nm redshift in excitation wavelengths†

**DOI:** 10.1039/d3sc02681g

**Published:** 2023-08-08

**Authors:** Jussi Isokuortti, Thomas Griebenow, Jan-Simon von Glasenapp, Tim Raeker, Mikhail A. Filatov, Timo Laaksonen, Rainer Herges, Nikita A. Durandin

**Affiliations:** a Faculty of Engineering and Natural Sciences, Tampere University FI-33101 Tampere Finland nikita.durandin@tuni.fi; b Otto-Diels-Institute of Organic Chemistry, Christian-Albrechts-University of Kiel 24098 Kiel Germany rherges@oc.uni-kiel.de; c Institute for Physical Chemistry, Department for Theoretical Chemistry, Christian-Albrechts-University of Kiel 24098 Kiel Germany; d School of Chemical and Pharmaceutical Sciences, Technological University Dublin, City Campus Grangegorman Dublin 7 Ireland; e Drug Research Program, Division of Pharmaceutical Biosciences, Faculty of Pharmacy, University of Helsinki Finland

## Abstract

Diazocines are bridged azobenzenes with phenyl rings connected by a CH_2_–CH_2_ group. Despite this rather small structural difference, diazocine exhibits improved properties over azobenzene as a photoswitch and most importantly, its *Z* configuration is more stable than the *E* isomer. Herein, we reveal yet another unique feature of this emerging class of photoswitches. In striking contrast to azobenzenes and other photochromes, diazocine can be selectively switched in *E* → *Z* direction and most intriguingly from its thermodynamically stable *Z* to metastable *E* isomer upon successive excitation of two different triplet sensitizers present in solution at the same time. This approach leads to extraordinary large redshift of excitation wavelengths to perform isomerization *i.e.* from 400 nm blue to 530 nm green light (*Z* → *E*) and from 530 nm green to 740 nm far-red one (*E* → *Z*), which falls in the near-infrared window in biological tissue. Therefore, this work opens up of potential avenues for utilizing diazocines for example in photopharmacology, smart materials, light energy harvesting/storage devices, and out-of-equilibrium systems.

## Abstract 2

Diazocine (bridged azobenzene) can be switched in both directions upon selective excitation of two different coexisting triplet sensitizers. Compared to direct irradiation, both switching wavelengths are strongly (≥130 nm) bathochromically shifted.

## Graphical abstract

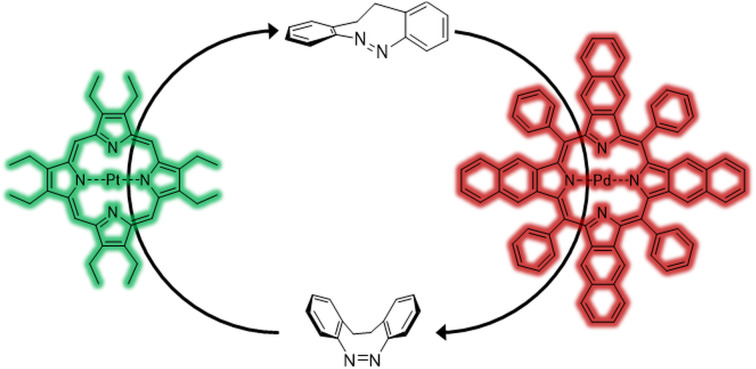

## Introduction

Diazocines, or bridged azobenzenes, have received considerable attention because their photoswitching behavior is inverted compared to conventional azobenzenes as their *Z* isomers are more stable than their *E* forms. Diazocines also exhibit more efficient photoswitching using longer excitation wavelengths and possess higher quantum yields.^1–13^ As a result, diazocines have found use in emerging fields, such as stimuli-responsive systems,^14–19^ photoactuation,^20^ and most notably, photopharmacology.^21–28^

**Scheme 1:**
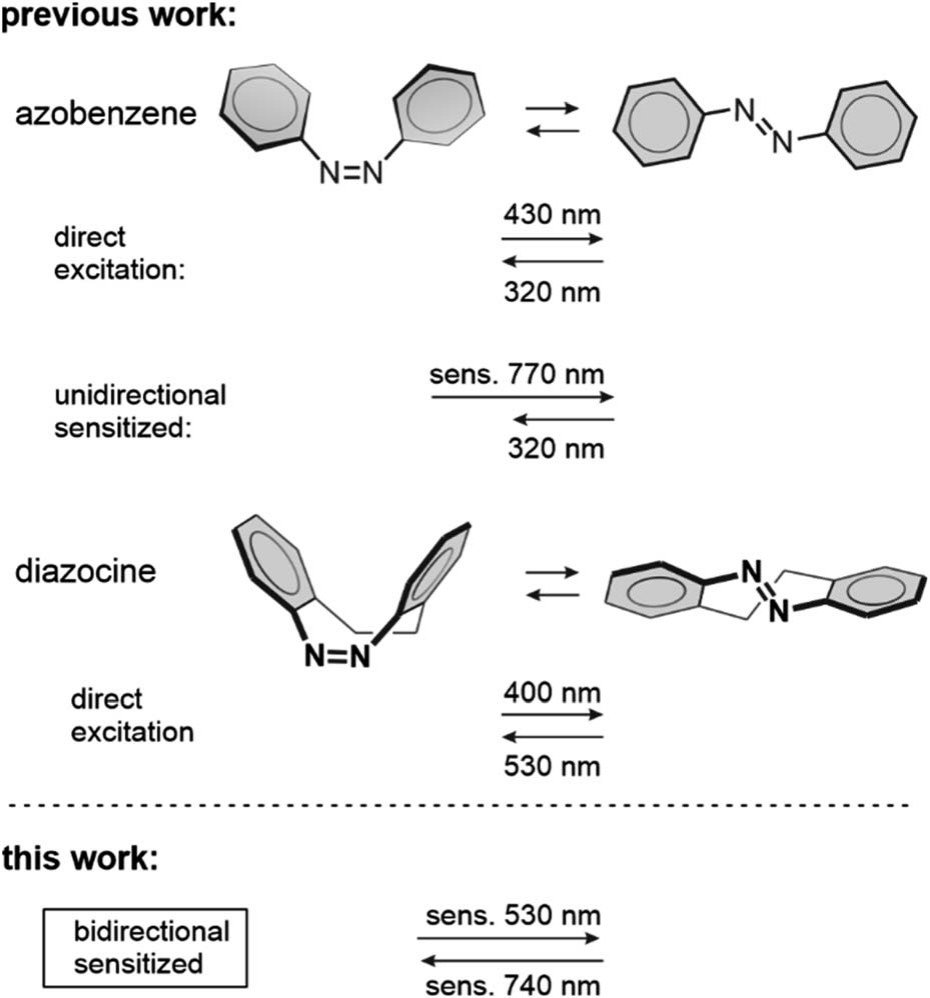
Direct and triplet-sensitized photoisomerizations of azobenzene and diazocine. The *Z* → *E* azobenzene isomerization is highly favored upon triplet sensitization in bulk.^38^ In this work, we report on the sensitized switching of diazocine in both directions (*Z* → *E* and *E* → *Z*). Compared to direct photoisomerization, both switching wavelengths are considerably redshifted.

Unlike conventional azobenzene, whose triplet state properties have been investigated since the 1960s,^29^ no studies on the triplet states of diazocine molecules have been published. Knowledge of the triplet state properties has been exploited to achieve sensitized photoswitching of conventional azobenzenes^30^ (Scheme 1) in addition to other classes of molecules, such as diarylethenes,^31,32^ overcrowded alkenes,^33^ stilbenes,^34,35^ indigos^36^ and norbornadienes.^37^ Compared to the direct excitation of the photoswitch, triplet sensitization enables photoswitching with red-shifted excitation wavelengths from the ultraviolet and blue, even towards near-infrared. This is paramount for most of the applications of photoswitching materials due to the harmful character and limited penetration depth of high energy photons. Thus, we embarked on investigating the triplet state properties of diazocine and uncovering whether triplet sensitization can improve its photoswitching performance.

## Results and discussion

**Fig. 1:**
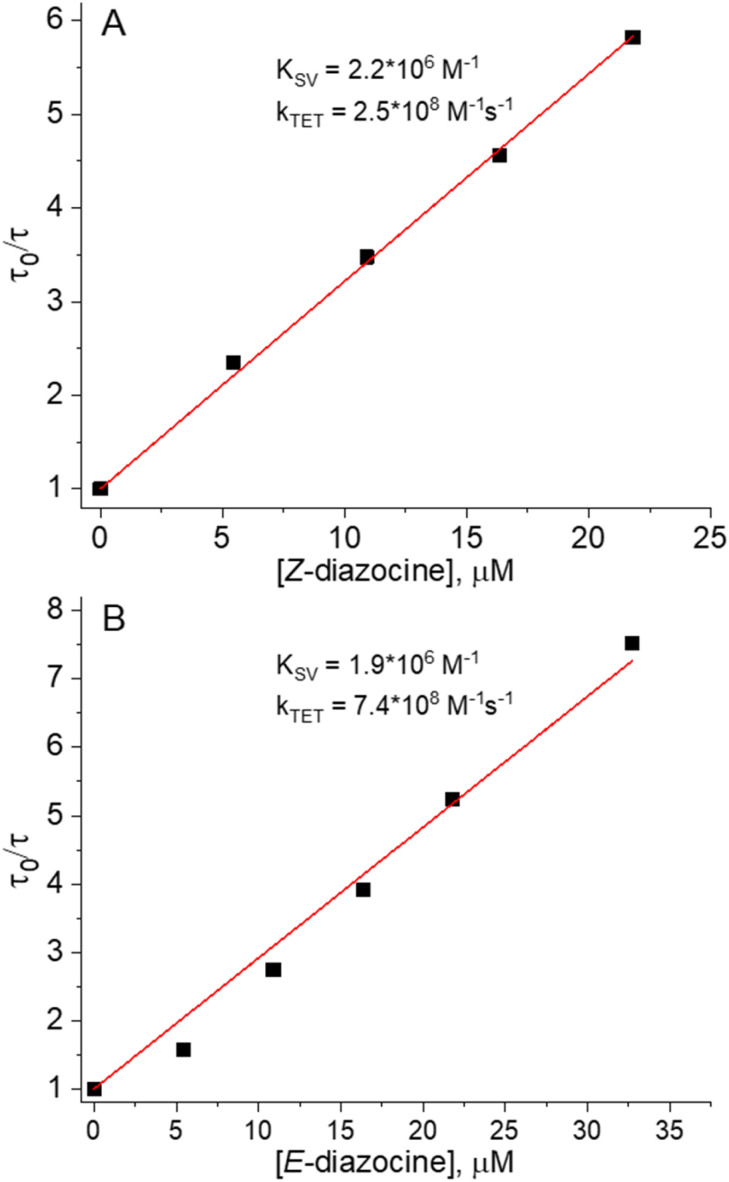
Phosphorescence quenching results of (A) PdOEP by *Z*-diazocine and (B) PdTPBP by *E*-diazocine as Stern–Volmer plots with linear fits for Stern–Volmer constants (*K*_SV_) and rate constants of triplet energy transfer (*k*_TET_).

**Chart 1:**
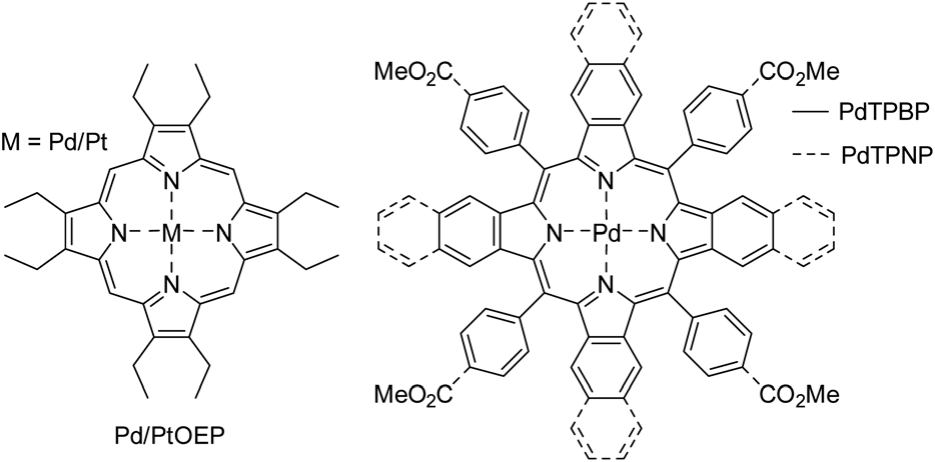
Triplet sensitizers used in this study.

We began investigating the diazocine triplet state properties by probing the triplet state energies (*E*_T_) of both isomers in deoxygenated DMSO. This was performed by measuring the triplet lifetime of a sensitizer (Chart 1) in the presence of varied concentrations of either *Z* or *E*-diazocine. The first quenching series was performed by quenching Pd(ii)-tetraphenyltetrabenzoporphyrin (PdTPBP, *E*_T_ = 1.55 eV, Fig. S1†) phosphorescence with *Z*-diazocine. We chose PdTPBP as the sensitizer as we expected *E*_T_ of *Z*-diazocine to be comparable to *E*_T_ of the *Z*-isomer of conventional azobenzenes, which have reported values between 1.3 and 1.6 eV.^39,40^ However, as [*Z*-diazocine] = 65 μM resulted in less than 20% quenching (Fig. S5 and S6†) of PdTPBP phosphorescence, it became clear that a more potent sensitizer was required as a triplet energy donor for *Z*-diazocine. Thus, we chose Pd(ii)-octaethylporphyrin (PdOEP, *E*_T_ = 1.86 eV, Fig. S2†) as another sensitizer for the second quenching series (Fig. S7†). The resulting Stern–Volmer plot is shown in Fig. 1A. Based on the Stern–Volmer constant (*K*_SV_) and rate constant of triplet energy transfer (*k*_TET_ = *K*_SV_/*τ*_0_, where *τ*_0_ is the unquenched triplet lifetime of the sensitizer), we calculated *E*_T_ of *Z*-diazocine to be 1.2 *k*_B_*T* or 31 meV larger than PdOEP at room temperature (RT).^41^ Thus, we derive that *E*_T_ of *Z*-diazocine is approximately 1.89 eV, which is considerably higher than *E*_T_ of conventional *Z*-azobenzenes (*vide supra*).

Having determined *E*_T_ for *Z*-diazocine, we turned our attention to *E*-diazocine and decided to begin the quenching studies again with PdTPBP. Before PdTPBP phosphorescence decay measurements, the *Z*-diazocine was converted into the *E*-isomer using 385 nm light irradiation for 10 minutes. Assuming again similar behavior as conventional azobenzenes that do not exhibit large difference in *E*_T_ between the isomers,^39,40^ we expected *E*-diazocine to also possess a high-lying triplet state and therefore poor energy transfer efficiency from PdTPBP. To our surprise, *E*-diazocine was able to quench the PdTPBP triplet state effectively (Fig. 1B and S8†) as we determine *E*_T_ of *E*-diazocine to be approx. 0.7 *k*_B_*T* or 18 meV (at RT) lower than that of PdTPBP *i.e.* 1.53 eV. As such, *E*_T_ of *E*-diazocine is comparable to that of the *E*-isomer of conventional azobenzenes.^40,42,43^ Conclusively, the triplet-state energies of *Z* and *E*-diazocine are energetically quite distinct, with the *Z*-isomer having a 360 meV higher triplet-state energy.

After determining the triplet energies of both isomers, we proceeded to photoswitching studies in deoxygenated DMSO with bis(methylthio)methane as an oxygen scavenger^44^ and [diazocine] = 500 μM. Initially, we paired diazocine again with PdTPBP. As expected, no *Z* → *E* isomerization was observed upon 640 nm excitation as *E*_T_ of *Z*-diazocine is over 300 meV higher than *E*_T_ of PdTPBP. Consequently, after first irradiating the system at 385 nm to drive direct *Z* → *E* isomerization of diazocine, rapid and complete *E* → *Z* isomerization was observed under red-light (640 nm) excitation (Fig. S9†). Although the absorption band of *E*-diazocine tails off beyond 600 nm, direct *E* → *Z* isomerization can still be driven with red-light excitation, an undisputed advantage over conventional azobenzene. However, pairing diazocine with PdTPBP affords photoswitching rates over twice as fast as those achieved with diazocine alone under red-light excitation. This rate enhancement is enabled by efficient triplet energy transfer from PdTPBP to *E*-diazocine and the more than two orders of magnitude higher molecular extinction coefficient of the sensitizer at 640 nm with respect to diazocine (Fig. S9†). Inspired by this improvement in the photoswitching performance, we were determined to push the limits of this system towards near infrared.

Conventional azobenzenes are known to undergo indirect *Z* → *E* photoisomerization even if the sensitizer *E*_T_ is substantially lower than the azobenzene.^40,45^ This is due to the ultrashort lifetime of azobenzene triplet state and thus negligible probability of back energy transfer to the sensitizer.^40^ This was also the case with *E*-diazocine, as Pd-tetraphenyltetranaphthoporphyrin (PdTPNP, *E*_T_ = 1.30 eV, Fig. S3†) can sensitize the *E* → *Z* isomerization under far-red 740 nm excitation (Fig. S10†), which is well within the bio-optical window. In this regard, the triplet state properties of diazocine are akin to azobenzene, as low triplet energy photosensitizers can drive the isomerization from the metastable isomer to the thermodynamically stable one.

To better comprehend the triplet state properties of diazocine, we performed photoswitching studies by sensitizing *Z*-diazocine with the higher triplet energy sensitizer PdOEP. Upon green-light excitation (530 nm), rapid *Z* → *E* isomerization was observed (Fig. S11†). This resulted in approximately 25% conversion to *E*-isomer, which is in stark contrast to conventional azobenzenes that exhibit approximately 1–2% conversion from the thermodynamically stable isomer upon sensitization.^39,42,43,46,47^ Consequently, the triplet-sensitized system also offers considerably red-shifted excitation wavelength from 402 nm to 534 nm (absorption maxima of *Z*-diazocine and PtOEP Q band, respectively) required for the isomerization when compared to the “photoswitch only” system.

After this exciting result, we sought to improve the *Z* → *E* conversion by changing the sensitizer from PdOEP to PtOEP as its higher-lying triplet state (*E*_T_ = 1.92 eV, Fig. S4†) enables more efficient energy transfer to *Z*-diazocine. This is crucial since the energy transfer to the lower triplet energy *E*-diazocine will begin to compete immediately as it is generated upon sensitization of *Z*-diazocine. Furthermore, the large difference between the triplet energies of the isomers indicated that they could be sensitized selectively with two different sensitizers present in the system.

**Fig. 2:**
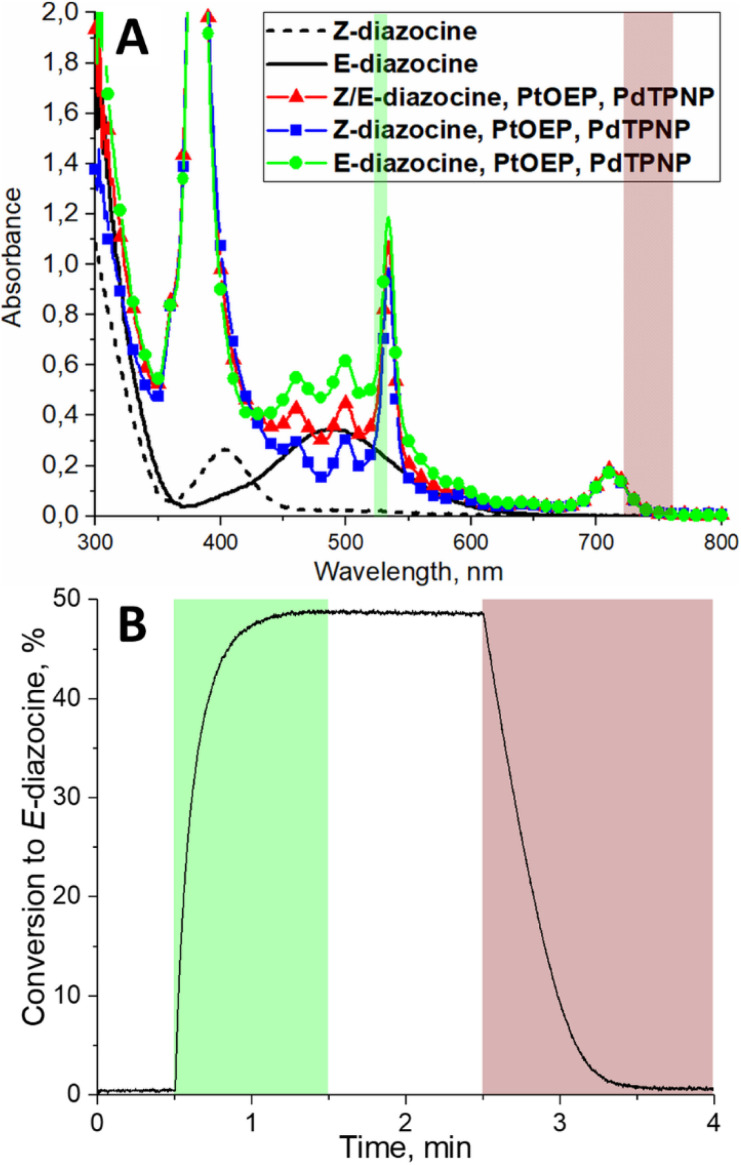
(A) Absorption spectra of *Z* and *E*-diazocine (after 385 nm excitation) and the photoswitching system consisting of diazocine, PtOEP and PdTPNP. The color bars indicate the excitation wavelengths to drive the triplet sensitized isomerization in both directions. (B) Diazocine photoswitching curve as the conversion percentage of *E*-diazocine monitored by absorption at 500 nm. The colored areas indicate time under excitation.

Thus, we decided to construct a photoswitching system consisting of three molecules: diazocine (500 μM), PtOEP (20 μM) and PdTPNP (1.2 μM) in deoxygenated DMSO. The absorption spectrum of this system is shown in Fig. 2A. Upon green-light (530 nm) excitation, the system reached 49% *Z*-to-*E* conversion, and upon 740 nm excitation, >99% *Z*-isomer (Fig. 2B). As discussed above, we attribute this more than twice improved *Z* → *E* conversion to more efficient triplet energy transfer to *Z*-diazocine from the higher triplet energy PtOEP. Noteworthy, due to lack of ground state coordination between *E*-diazocine and the porphyrins no increase in *E*-to-*Z* diazocine isomerization rate was observed in the dark (Fig. S12†). All in all, this is, to the extent of our knowledge, the first demonstration of a photoswitching system where no direct excitation of the photoswitch itself is required for bidirectional isomerization.

To gain more insight into the photophysical properties of diazocine, we performed quantum chemical calculations at the TDDFT and CASPT2 level of theory with ORCA 5.0.1, Turbomole 7.4 and OpenMolcas (for details, basis sets and active space, please, see ESI†).^48–53^ State averaged (SA) complete active space self-consistent field (CASSCF) and second order perturbation theory CAS (CASPT2) methods are known to provide reliable energies of high spin systems and particularly CASPT2 has emerged as the standard method to calculate excited state properties.^54^

The CASPT2 energies of the T_1_ and S_1_ excited states vertical to the S_0_ ground state of azobenzene and diazocine in *Z* and *E* configuration are given in Table 1. The calculated triplet energies (T_1_) are consistently about 0.3 eV higher than the measured values, which is within the usual range of accuracy.^55–57^ Both theoretical calculations and experiments predict similar triplet excitation energies for *Z* and *E* azobenzene (ΔΔ*E*_*E*–*Z*(calc.)_ = 0.04 eV, ΔΔ*E*_*E*–*Z*(exp.)_ = 0.03–0.27 eV). In contrast, the triplet energies of diazocine in *Z* and *E* configuration differ considerably (Table 1). The calculated and measured energy differences are in very good agreement (ΔΔ*E*_*E*–*Z*(calc.)_ = 0.38 eV, ΔΔ*E*_*EZ*(exp.)_ = 0.36 eV, Table 1). Computationally less expensive TDDFT calculations confirm the trend for azobenzene (ΔΔ*E*_*EZ*_ = 0.09 eV) and diazocine (ΔΔ*E*_*E*–*Z*_ = 0.35 eV).

**Table 1:** Experimentally determined and theoretically calculated vertical (diabatic) triplet and singlet excitation energies (eV) of azobenzene and diazocine, in *E* and *Z* configurations. For computational details, see ESI

	TDDFT	CASPT2	Exp.
**Azobenzene**			
*ΔE S_0_ → T_1_*			
Z	1.99	1.83	1.30–1.6 (ref. 39, 40, 42 and 43)
*E*	2.08	1.87 (ref. 55 and 56)	1.57 (ref. 39, 40, 42 and 43)
*ΔE S_0_ → S_1_*			
Z	2.98	2.58	2.82
*E*	2.85	2.44	2.78

**Diazocine**			
*ΔE S_0_ → T_1_*			
Z	2.09	2.21	1.89
*E*	1.74	1.83	1.53
*ΔE S_0_ → S_1_*			
Z	3.19	3.02	3.07
*E*	2.64	2.52	2.53 (ref. 10)

**Fig. 3:**
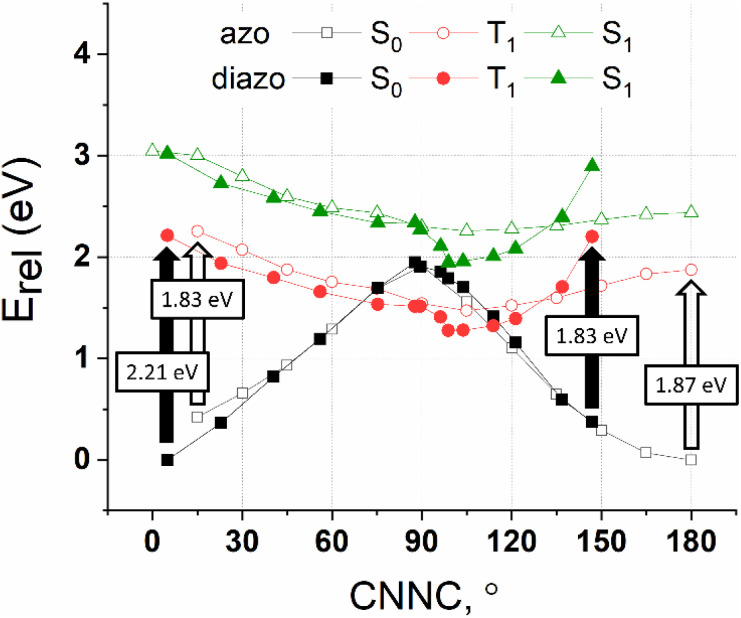
CASPT2 potential energies of azobenzene (“azo”) and diazocine (“diazo”) in their S_0_, T_1_ and S_1_ states as a function of the CNNC dihedral angle. Vertical transitions (S_0_ → T_1_) are indicated with arrows (filled for diazocine). Energies of azobenzene and diazocine are relative to their most stable ground state (S_0_) isomers (*E* azobenzene and *Z* diazocine). Note that the CNNC torsion in *Z* azobenzene is ∼12° because of the non-planarity caused by steric hindrance of the two phenyl rings. The CNNC torsion in *E* diazocine deviates strongly from 180° (147°) due to the large strain in the *trans* configured eight-membered diazocine ring. Energy scans, therefore, are restricted to the range of twist angles between the corresponding ground state minima (*E* and *Z*).

The CASPT2 calculated potential energy surface of the S_0_, T_1_ and S_1_ states of azobenzene and diazocine as a function of the CNNC twist angle are depicted in Fig. 3. Between 40° and 140° the energy surfaces of azobenzene and diazocine are almost identical. On the “*Z* side” (0–40°) and on the “*E* side” (140–180°) the energies differ. Azobenzene in its ground state (S_0_) is not planar but slightly twisted into the “*E* direction” (CNNC ∼ 12°) due to the steric repulsion of the phenyl rings.^58^ In contrast, the *E* configuration of diazocine is strongly distorted towards the “*Z*” form because of the high ring strain in the *E*-configured eight-membered diazocine ring (CNNC = 147° instead of 180°).

## Conclusions

In summary, we have succeeded for the first time in bidirectional switching a photochromic compound with two different triplet sensitizers present at the same time. The molecular photoswitch diazocine isomerizes upon direct irradiation at 400 nm (*Z* → *E*) and 530 nm (*E* → *Z*). Upon indirect, triplet-sensitized excitation, the switching wavelengths redshift to 530 and 740 nm, respectively. The *E* → *Z* isomerization can thus be achieved with far-red light well within the bio-optical window. This is advantageous for both biological and medical applications. Even more interesting is that indirect electronic excitation permits additional control over the spatial addressing and the direction of the switching process. For example, upon direct irradiation with green light, an *E* → *Z* isomerization is induced, and in the presence of a suitable triplet sensitizer (PtOEP), the process is reversed, which could be utilized for controlled chemical energy storage and release. Likewise, by employing two different sensitizers, as demonstrated herein, even more sophisticated systems can be realized.

## Data availability

Original data used to plot figures of the main text have been made publicly available *via* Zenodo and can be found at: https://doi.org/10.5281/zenodo.8238992.

## Author contributions

JI: performed phosphorescence and photoswitching studies. TG: prepared the diazocine and determined the photophysical data. JSvG: performed geometry optimizations TDFT calculations. TR: performed CASCF and CASPT2 calculations. MAF: synthesized and characterized Pd-tetraphenyltetranaphthoporphyrin. TL: acquired funding, supervised the work done in Tampere University. RH: supervised diazocine synthesis and calculations. NAD: performed steady-state absorption measurements and supervised the work done in Tampere University. All authors discussed and commented on the manuscript.

## Conflicts of interest

There are no conflicts to declare.

## Supplementary Material

SC-014-D3SC02681G-s001
