# Supplementary material for: Triplet sensitization enables bidirectional isomerization of diazocine with 130 nm redshift in excitation wavelengths
Source: Chem Sci. 2023 Aug 8;14(34):9161–6. doi: 10.1039/d3sc02681g (PMC10466275; doi:10.1039/d3sc02681g)
Supplement: SC-014-D3SC02681G-s001 [file SC-014-D3SC02681G-s001.pdf]

Electronic Supplementary Material (ESI) for Chemical Science.
This journal is © The Royal Society of Chemistry 2023

Supporting Information

Triplet Sensitization Enables Bidirectional Isomerization of Diazocine with 130 nm Redshift in Excitation Wavelengths

Jussi Isokuortti[a], Thomas Griebenow[b], Jan-Simon von Glasenapp[b], Tim Raeker[c], Mikhail A. Filatov[d], Timo Laaksonen[a,e], Rainer Herges[b*], Nikita A. Durandin[a*]

[a.] *Faculty of Engineering and Natural Sciences, Tampere University, FI-33101 Tampere, Finland. Email: nikita.durandin@tuni.fi*
[b.] *Otto-Diels-Institute of Organic Chemistry, Christian-Albrechts-University of Kiel, 24098 Kiel, Germany. Email: rherges@oc.uni-kiel.de*
[c.] *Institute for Physical Chemistry, Department for Theoretical Chemistry, Christian-Albrechts-University of Kiel,24098 Kiel, Germany*
[d.] *School of Chemical and Pharmaceutical Sciences, Technological University Dublin, City Campus, Grangegorman, Dublin 7, Ireland*
[e.] *Drug Research Program, Division of Pharmaceutical Biosciences, Faculty of Pharmacy, University of Helsinki*

Table of Contents

Materials 2
Triplet energies of the sensitizers 2
Phosphorescence quenching experiments 4
Isomerization experiments 8
Computational details 12
  DFT and TD-DFT 12
  CASSCF/CASPT2 15
Synthesis of 11,12-Dihydrodibenzo[c,g][1,2]diazocine 17
References 18

Materials

All the solvents were purchased from Sigma-Aldrich (Merck KGaA, Germany) and used as provided. PdTPBP, PdOEP and PtOEP were purchased from Frontier Scientific Inc. (USA). The synthesis of PdTPNP[1] was previously published. Diazocine was synthesized according to a modified procedure (detailed and characterized below) published by Moormann et al.[18] Bis(methylthio)methane was purchased from Tokyo Chemical Industry (Japan).

Triplet energies of the sensitizers

Phosphorescence spectra and phosphorescence decays for quenching studies were measured with FLS1000 spectrofluorometer (Edinburgh Instruments Ltd, UK). Phosphorescence samples were prepared in a 1 cm^2 SOG9 cuvette (Starna Scientific Ltd, UK) sealed with a screw cap and a silicon/PTFE septum. 1 µM concentration of sensitizer was dissolved in DMSO to which 0.48 M concentration of Bis(methylthio)methane was added. The sample was then purged of oxygen by bubbling vigorously with nitrogen for 1 hour. The phosphorescence spectra (Fig. S1-S4) were measured by exciting the sensitizers at their Q band maxima with a Xe lamp. Their triplet energies were then determined by the maxima wavelengths.

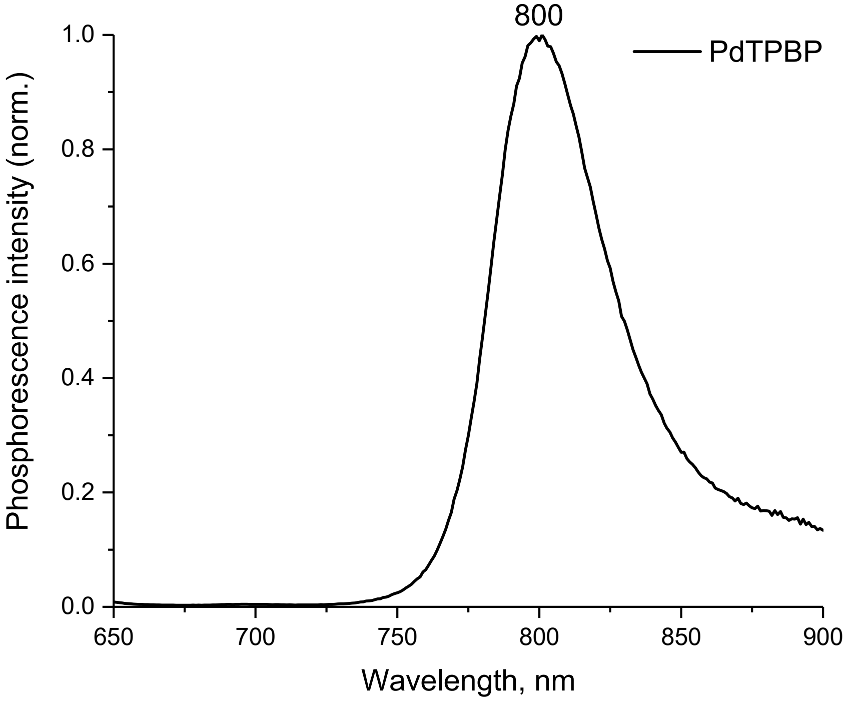

Figure S1. Normalized phosphorescence spectrum of PdTPBP. The wavelength of the maximum is labeled.

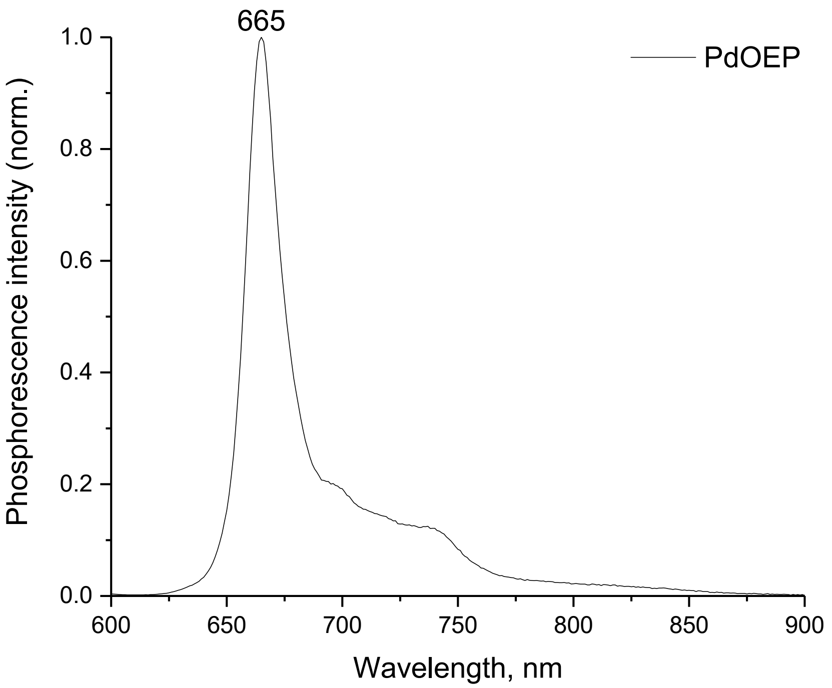

Figure S2. Normalized phosphorescence spectrum of PdOEP. The wavelength of the maximum is labeled.

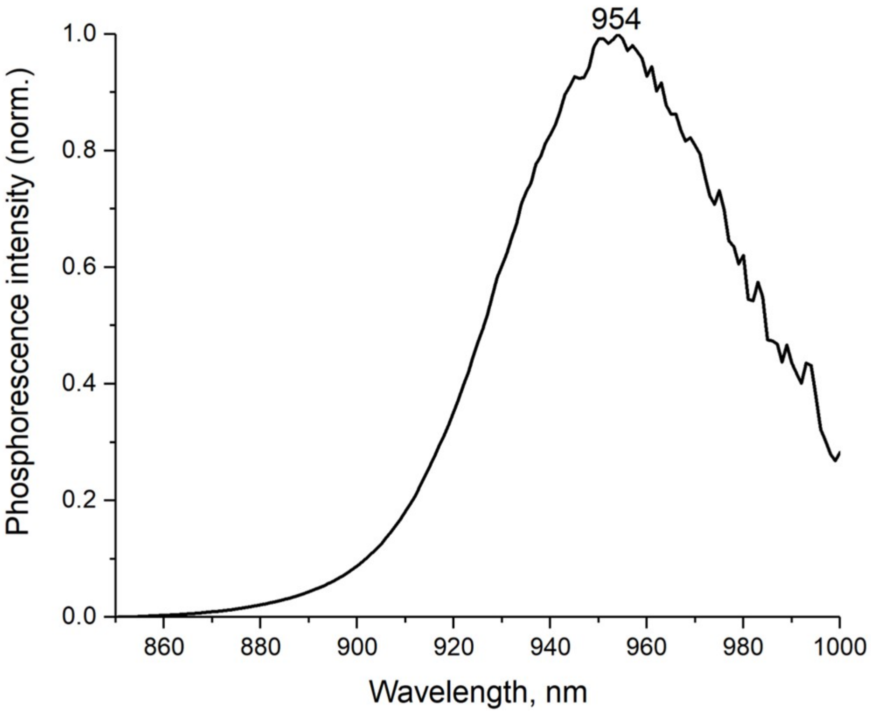

Figure S3. Normalized phosphorescence spectra of PdTPNP. The wavelength of the maximum is labeled.

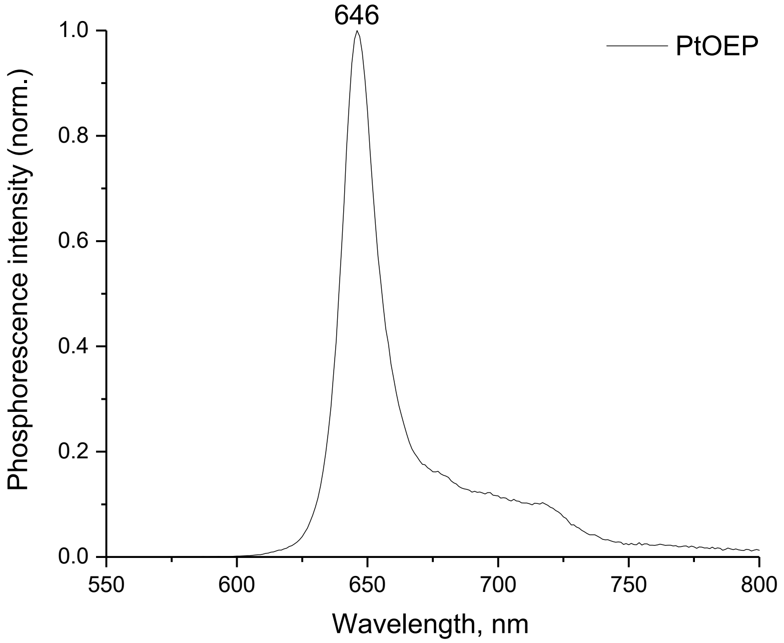

Figure S4. Normalized phosphorescence spectrum of PtOEP. The wavelength of the maximum is labeled.

Phosphorescence quenching experiments

Quenching samples were prepared as described above with 1 μM concentration of sensitizer. Diazocine was added as chloroform solution to the sample and purged for 1 hour after each addition before measuring the phosphorescence decays. The quenching with diazocine in *E*-conformation was performed by illuminating the sample by 385 nm excitation with the xenon lamp of the spectrofluorometer for 15 min. The resulting phosphorescence decays were then tail-fitted to extract the phosphorescence lifetimes. All decays were monoexponential ($\chi < 1.2$). The phosphorescence decays of each quenching pair are shown in Figures S5, S7, S8. A linear equation was fitted ($R^2 > 0.99$) on Origin 2017 on the Stern-Volmer plots. The monoexponential decays and linearity of the plots Stern-Volmer plots indicates that nearly pure (>90 %) *Z* or *E* isomer was present in the samples. The diffusion rate constant in DMSO was evaluated by performing phosphorescence quenching of PdTPBP with 9,10-bis(phenylethynyl)anthracene that has triplet energy of 1.12-1.30 eV[2]. The large exothermic energy gap (≥ 0.25 eV or 10 k_BT) between the triplet donor and acceptor thus ensures truly diffusion-controlled rate for triplet energy transfer, i.e. $k_{TET} = k_{diff} \approx 1.1 \times 10^9\ M^{-1}s^{-1}$ in DMSO.

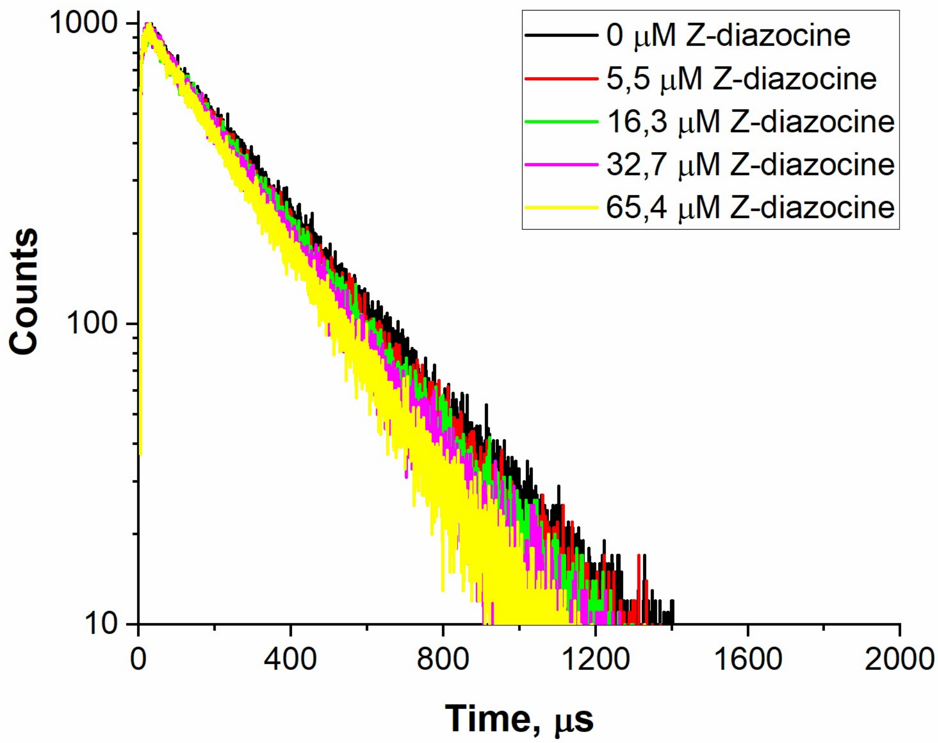

Figure S5. Phosphorescence decays of PdTPBP in presence of *Z*-diazocine.

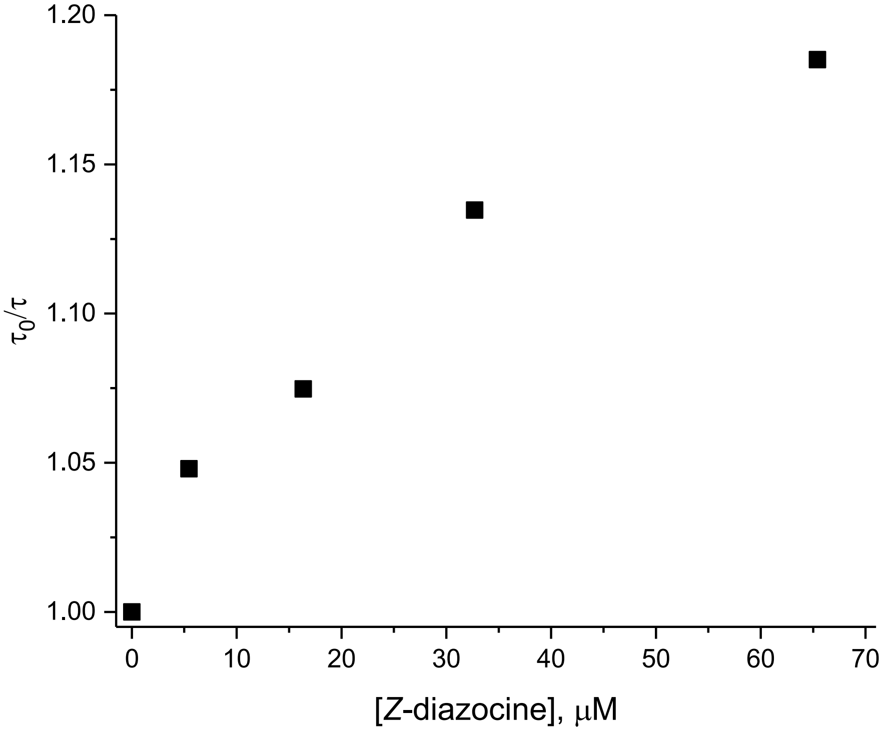

Figure S6. Quenching results of PdTPBP phosphorescence by *Z*-diazocine as a Stern-Volmer plot.

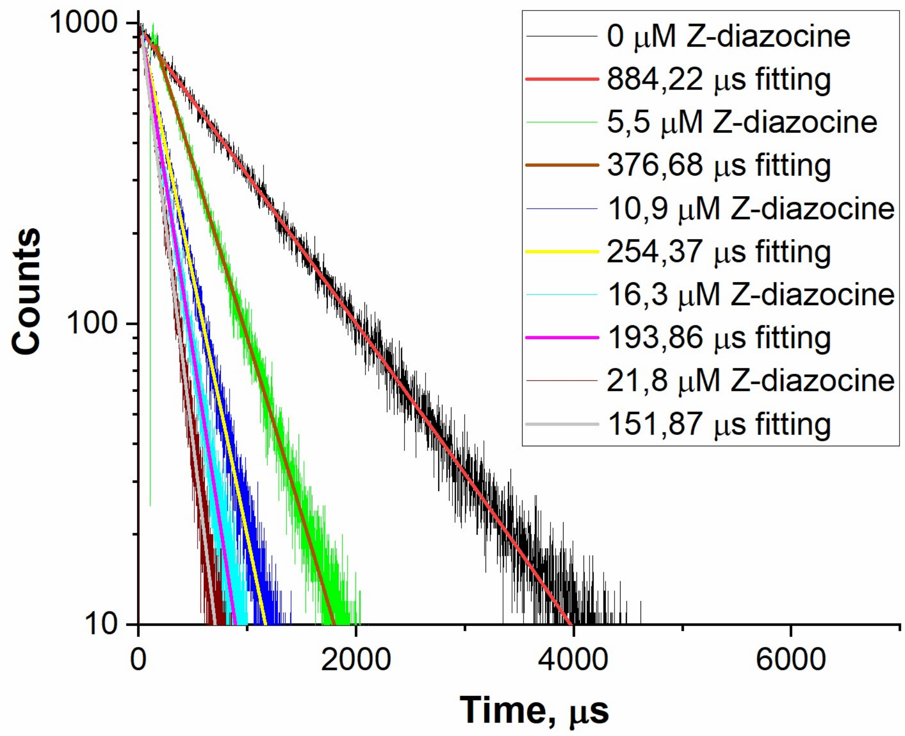

Figure S7. Phosphorescence decays of PdOEP in presence of *Z*-diazocine.

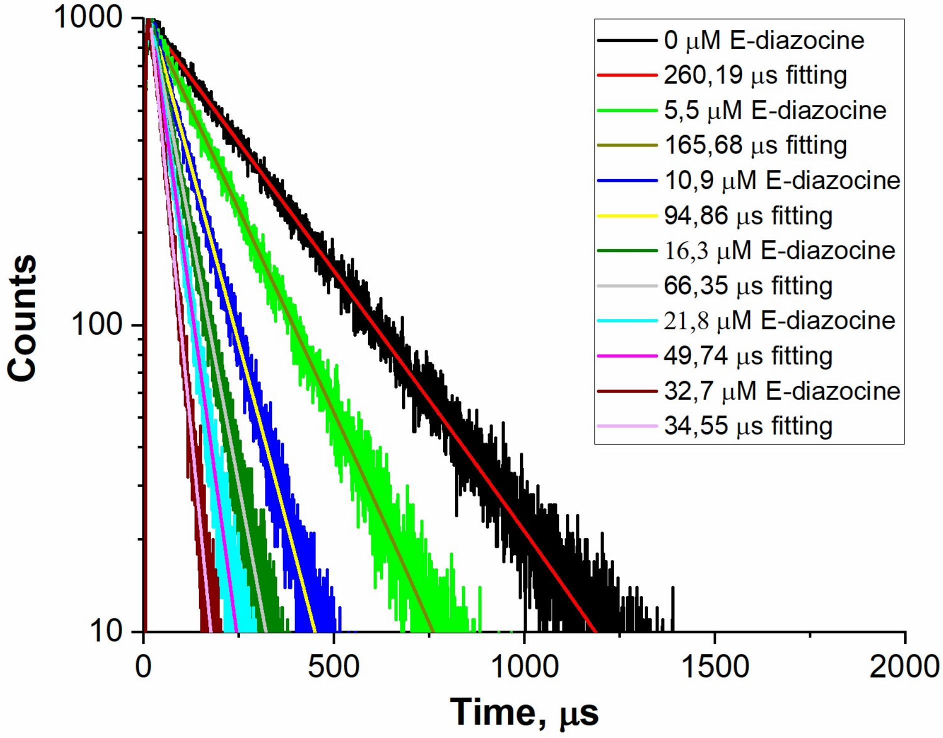

Figure S8. Phosphorescence decays of PdTPBP in presence of *E*-diazocine.

Isomerization experiments

Photoisomerization studies were performed also in DMSO to which 0.48 M concentration of Bis(methylthio)methane was added. The concentration of diazocine was constant, 500 µM, for all isomerization studies. The samples were also prepared in screw cap cuvettes (vide supra) and purged vigorously with nitrogen for 1 h prior to measuring.

The photoisomerization measurements were performed on a Cary 60 spectrophotometer (Agilent Technologies Inc, USA) equipped with an Ocean Optics (USA) qpod 2e Peltier-thermostated cell holder at 20 ℃. Excitation source was Prior Lumen 1600 (Prior Scientific Inc, USA) with multiple choices for narrow-band LEDs at different wavelengths. Direct excitation of *Z*-diazocine was performed with a 385 nm LED (230 mW/cm^2), excitation of PdOEP or PtOEP was performed with a 530 nm LED (140 mW mW/cm^2), excitation of PdTPBP was performed with 640 nm LED (255 mW/cm^2) and excitation of PdTPNP was performed with a 740 nm LED (255 mW/cm^2). The conversion between *Z*- and *E*-isomers was monitored by absorption at 500 nm.

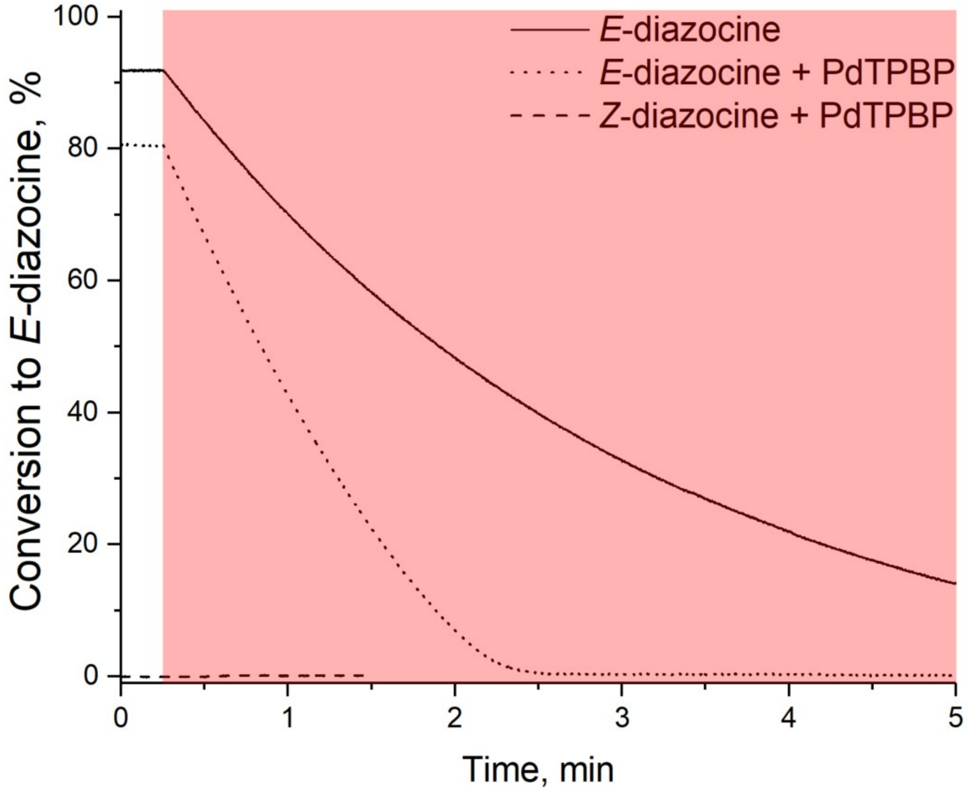

Figure S9. Isomerization curves of diazocine and diazocine + PdTPBP (1.2 μM) under 640 nm excitation. *E*-diazocine samples were first illuminated with 385 nm excitation to drive *Z*-to-*E* isomerization (not shown).

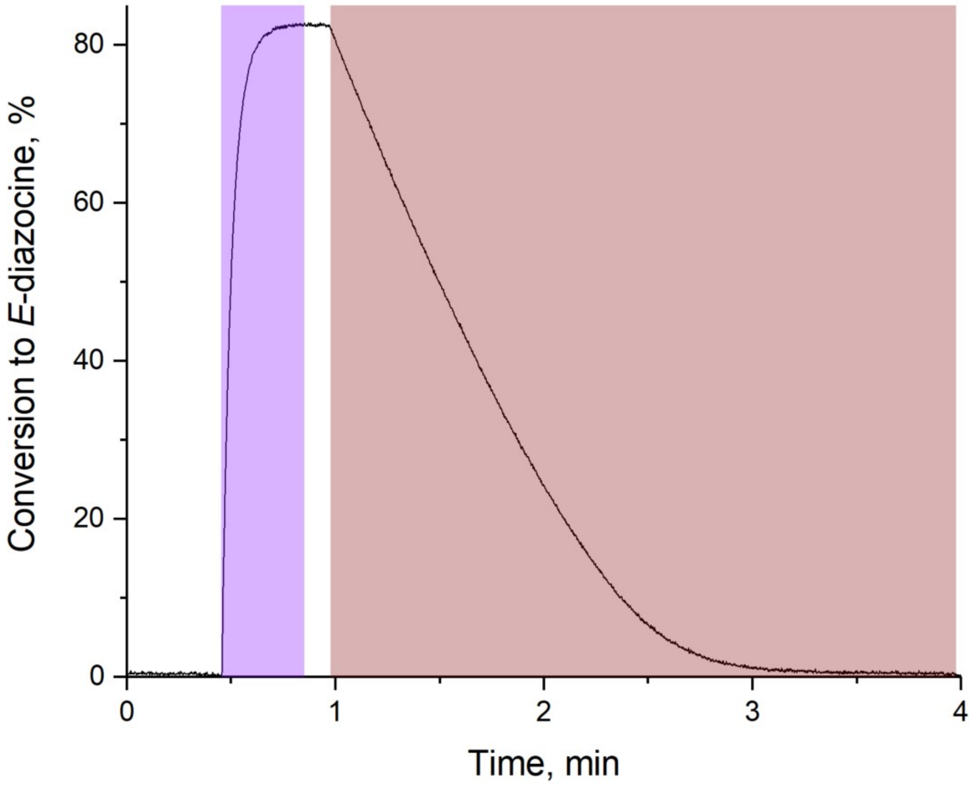

Figure S10. Isomerization curve of diazocine + PdTPNP (1.2 μM) under 385 nm (*Z*-to-*E* isomerization) and 740 nm (*E*-to-*Z* isomerization) excitation. The color bars indicate time under excitation.

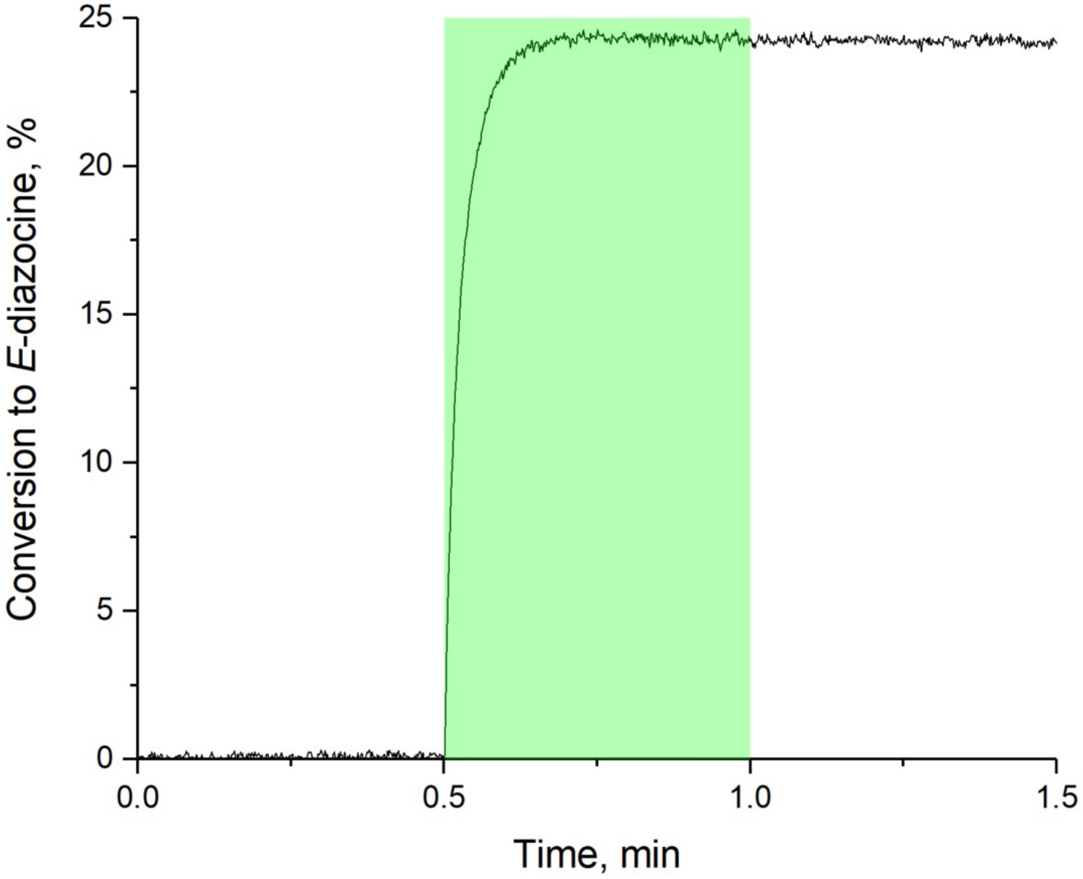

Figure S11. Isomerization curve of diazocine + PdOEP (20 μM) under 530 nm (*Z*-to-*E* isomerization). The color bar indicates time under excitation.

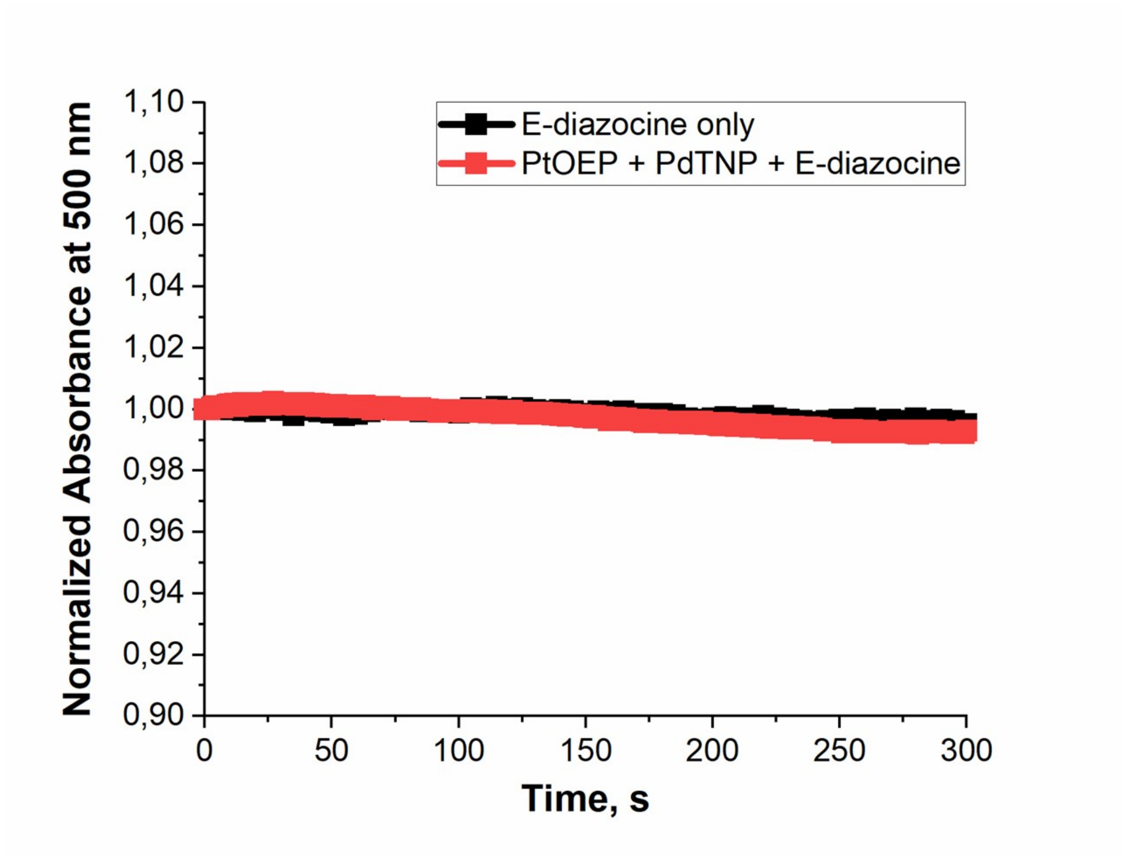

Figure S12. Comparison of *E*-to-*Z* isomerization of Diazocine in the dark with and without porphyrin sensitizers.

Computational details

DFT and TD-DFT

TD-DFT calculations were carried out on a ωB97X-D3/def2-TZVP[4,5] level of theory using ORCA[6] with Grimmes D3 dispersion correction[7] and counter poise corrections for basis set superposition errors (BSSE).[8] Preliminary scans along the CNNC torsion were carried out for diazocine and azobenzene (AB), all other coordinates were allowed to relax freely. S_1 and T_1 energies were determined for vertical excitation starting from the respective S_0 geometry. Unfortunately, the CNNC torsional angles become ill-defined between 70° and 110° because one of the NNC angles is close to 180° (inversion mechanism). Vertical excitations of the *E* and *Z* isomers obtained with TD-DFT are shown in Table S1.

Azobenzene	ωB97X-D3/def2-TZV	
	Z	*E*
ΔE $S_0 \rightarrow S_1$	2.98	2.85
ΔE $S_0 \rightarrow T_1$	1.99	2.08
Diazocine		
	Z	*E*
ΔE $S_0 \rightarrow S_1$	3.19	2.64
ΔE $S_0 \rightarrow T_1$	2.09	1.74

Table S1 $S_0 \rightarrow S_1$ and $S_0 \rightarrow T_1$ Energy gaps obtained on a ωB97X-D3/def2-TZVP level of theory. All energies in eV.

To circumvent the problem of ill-defined CNNC torsion angles we choose to use a nudged elastic band approach (NEB)[9] to determine the minimum energy pathway (MEP) from the diazocine *Z* to the *E* isomer. After the minimum energy pathway was determined we calculated the vertical excitations from the obtained S_0 geometries. The S_0 MEP of the diazocine isomerization, as well as S_1 and T_1 directly above this path are shown in Figure S9. The corresponding MEP of azobenzene is shown in Figure S10.

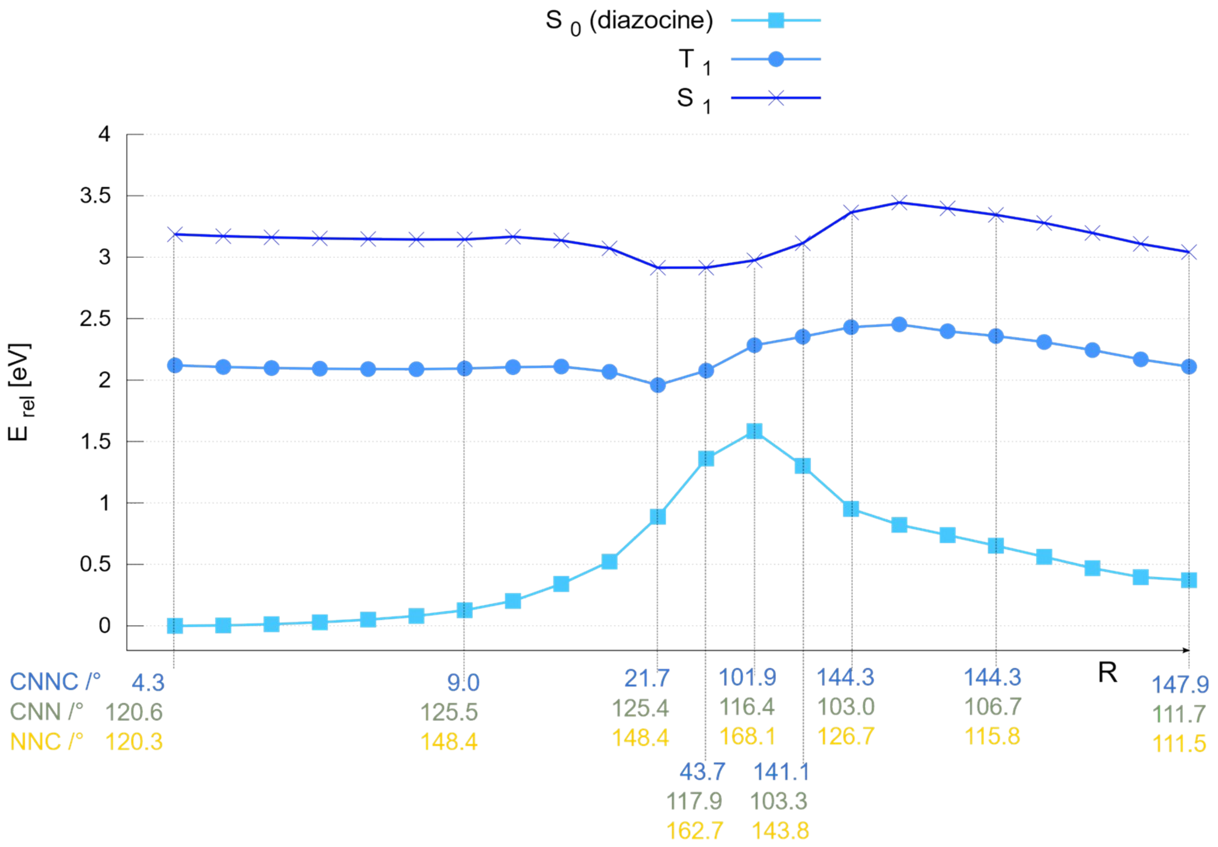

Figure S13. MEP of the diazocine isomerization at the ωB97X-D3/def2-TZVP level of time dependent density functional theory (TDDFT) with vertical excitations to S_1 and T_1. Relative energies are in eV. The reaction coordinate R is defined by the nudged band (NEB) approach. The stationary points (*Z* and *E* isomers and the transition state) at the S_0 energy hypersurface are fully optimized.

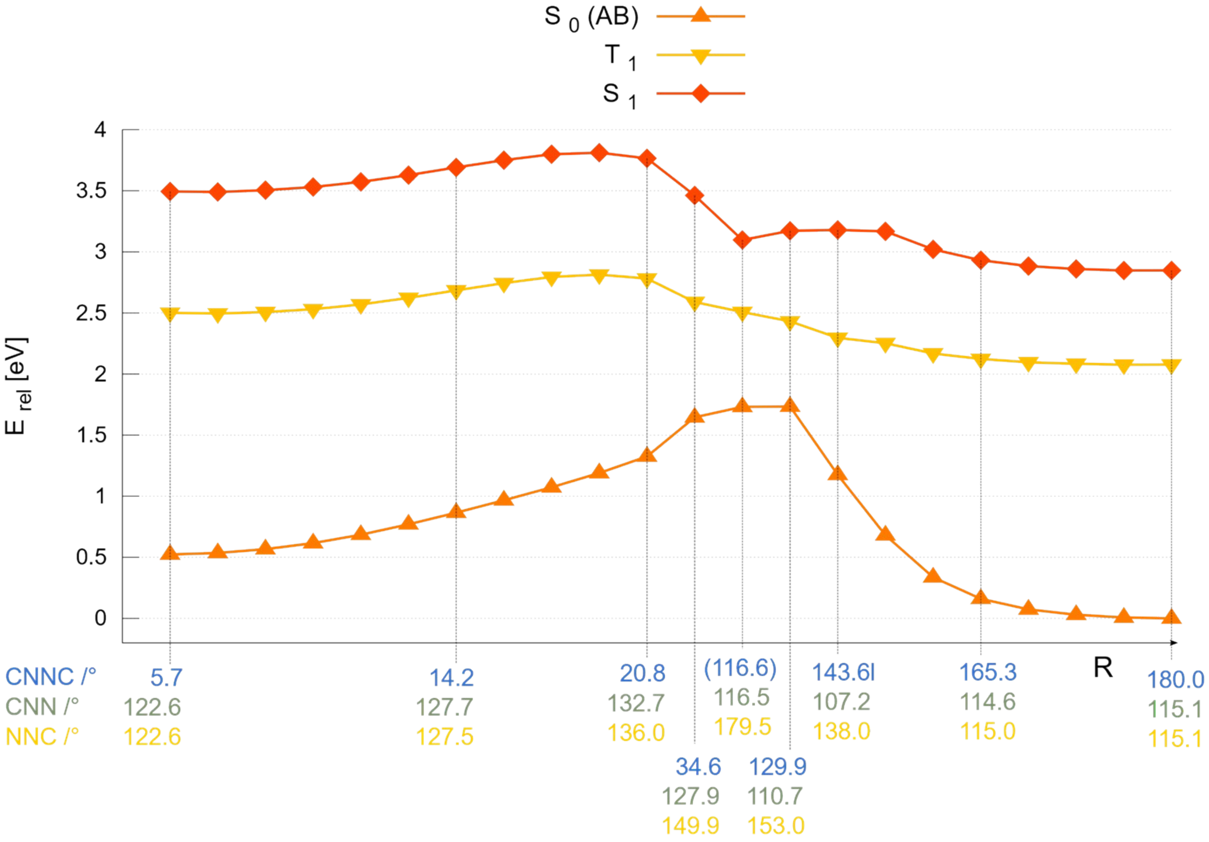

Figure S14. MEP of the azobenzene isomerization at the ωB97X-D3/def2-TZVP level of time dependent density functional theory (TDDFT) with vertical excitations to S_1 and T_1. Relative energies are in eV. The reaction coordinate R is defined by the nudged band (NEB) approach. The stationary points (*Z* and *E* isomers and the transition state) at the S_0 energy hypersurface are fully optimized.

CASSCF/CASPT2

The state-averaged (SA) complete active space self-consistent field (CASSCF) and second order perturbation theory CAS (CASPT2) calculations were carried out with the OpenMolcas program package (version 22.06)[10]. For both azobenzene and diazocine, we adopted most of the computational setup from a previous work[11]: The active space consists of 14 electrons in the 12 relevant n, π and π* orbitals; different atoms were treated with different basis sets (Dunning's aug-cc-pVTZ basis set for the two nitrogen atoms and cc-pVTZ for all carbon atoms [12] and Pople's STO-3G for the H atoms [13]; Cholesky decomposition was enabled with the 'medium' keyword. The structures for diazocine were also taken from[11]. For azobenzene a simple scan around the central N=N bond in 15 steps resulted in 13 structures which were optimized using D4-RI-B3LYP/def2-SVP [4,14-16] with Turbomole v7.4 [17].The following procedure was then applied to all structures: SA-CASSCF energy calculations for the first three electronic singlet states followed by

the corresponding CASPT2 energy calculation; both CASSCF and CASPT2 energy calculations for the first triplet state; calculation of the spin-orbit coupling (SOC) on the CASPT2 level.

Table S2 lists all S_0, T_1, S_1 and S_2 energies on the CASPT2 level relative to the lowest energy in the electronic ground state for both diazocine and azobenzene. The SOC calculation turned out to be not necessary as all relative energies are virtually the same compared to the "pure" CASPT2 energies. All energies in eV.

azobenzene					diazocine				
CNNC/°	S_0	T_1	S_1	S_2	CNNC/°	S_0	T_1	S_1	S_2
0	1.4872	2.449	3.0462	5.7216	4.946	0	2.2121	3.0198	4.722
15	0.4236	2.2564	3.0006	5.2008	22.891	0.3638	1.9386	2.7252	4.5008
30	0.6571	2.0709	2.7936	4.8127	40.479	0.8204	1.7956	2.5835	4.4103
45	0.9336	1.8764	2.5976	4.2915	56.016	1.1915	1.6571	2.4494	3.9748
60	1.2895	1.7543	2.4866	3.8122	75.305	1.6959	1.5349	2.3367	3.2799
75	1.687	1.6889	2.4339	3.3461	87.655	1.9497	1.5141	2.3425	2.9059
90	1.9102	1.542	2.2987	2.776	89.452	1.9072	1.5149	2.2727	3.1566
105	1.5568	1.4724	2.2555	2.9508	96.288	1.8573	1.4081	2.1082	3.0618
120	1.1049	1.5224	2.2777	3.4574	98.877	1.7901	1.2769	1.947	2.5054
135	0.6485	1.5971	2.3076	3.9308	103.773	1.7023	1.2791	1.9593	2.5988
150	0.2933	1.7189	2.3694	4.3568	113.894	1.4144	1.323	2.0085	3.023
165	0.0688	1.8328	2.4223	4.7004	121.291	1.1548	1.3931	2.08	3.4157
180	0	1.8739	2.438	4.9647	136.913	0.5919	1.7064	2.3944	4.5122
					146.926	0.3734	2.2019	2.8937	5.05

The essential $S_0 \rightarrow S_1$ and $S_0 \rightarrow T_1$ energy differences are compiled in Table S2. There, we also list the intermediate CASSCF and final CASPT2+SOC energies obtained during our calculations for the sake of completeness.

Table S3 Essential $S_0 \rightarrow S_1$ and $S_0 \rightarrow T_1$ energy differences All energies in eV.

azobenzene	CASSCF		CASPT2		CASPT2+SOC	
	Z	*E*	*Z*	*E*	*Z*	*E*
$S_0 \rightarrow S_1$	3.06	2.84	2.58	2.44	2.58	2.44
$S_0 \rightarrow T_1$	2.09	2.18	1.83	1.87	1.83	1.87
**diazocine**	**CASSCF**		**CASPT2**		**CASPT2+SOC**	
	Z	*E*	*Z*	*E*	*Z*	*E*
$S_0 \rightarrow S_1$	3.54	2.89	3.02	2.52	3.02	2.52
$S_0 \rightarrow T_1$	2.49	1.96	2.21	1.83	2.21	1.83

Synthesis of 11,12-Dihydrodibenzo[c,g][1,2]diazocine[18]

To a solution of 2,2'-dinitrodibenzyl (2.03 g, 7.5 mmol) in 400 mL EtOH was added an aqueous solution of $Ba(OH)_2$*8 H_2O (6.99 g, 22.2 mmol) in 200 mL H_2O and zinc powder (15.21 g, 232.7 mmol), and the mixture was stirred for 18 h under reflux. The reaction mixture was filtered through celite and the solvent was removed under reduced pressure. The crude product was dissolved in CH_2Cl_2 and filtered through celite, dried over $MgSO_4$, and the solvent was removed under reduced pressure. The crude product was dissolved in 500 mL methanolic NaOH solution, $CuCl_2$ was added, and air was bubbled through the solution while stirring at room temperature until completion of the reaction. The reaction mixture was neutralized with 1 M HCl solution. After addition of saturated sodium bicarbonate solution, the aqueous layer was extracted with CH_2Cl_2 three times. The combined organic layers were dried over $MgSO_4$ and the solvent was removed under reduced pressure. The crude product was purified by column chromatography (cyclohexane/EtOAc 3:1) on silica gel to afford the product as a yellow solid.

Yield: 833 mg (4 mmol, 54%) Lit.[18]: 58%

Melting point: 110 °C Lit.[18]: 103-105 °C

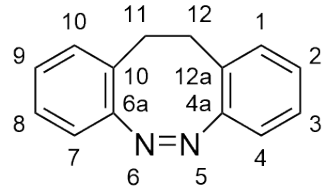

^{1}H-NMR (500 MHz, Acetone-D$_6$ 300 K): δ = 7.13 (td, ^3J = 7.5 Hz, ^4J = 1.6 Hz, 2 H, *H*-3, *H*-8), 7.03-6.96 (m, 4 H, *H*-1, *H*-10, *H*-2, *H*-9), 6.83 (dd, ^3J = 7.8 Hz, ^4J = 1.1 Hz, 2 H, *H*-4, *H*-7), 2.87 (m$_c$, 4 H, *H*-11, *H*-12) ppm.

^{13}C-NMR (125 MHz, Acetone-D$_6$ 300 K): δ = 155.46 (*C*-4a, *C*-6a), 129.61 (*C*-1, *C*-10), 128.08 (*C*-10a, *C*-12a), 127.02 (*C*-2, *C*-9), 126.67 (*C*-3, *C*-8), 118.70 (*C*-4, *C*-7), 31.68 (*C*-11, *C*-12) ppm.

MS (EI, 70 eV): *m/z* (%) = 208.10 (24) $[M]^+$, 180.09 (34) $[C_{14}H_{12}]^+$, 179.08 (100) $[C_{14}H_{11}]^+$, 178.07 (96) $[C_{14}H_{10}]^+$.

MS (HR): *m/z* ($C_{14}H_{12}N_2$) = calc.: 208.10005, found: 208.10010 ± 0.26 ppm.

IR (ATR): *ν* (cm^{-1}): 3058 (w), 2984 (w), 1813 (w), 1568 (w), 1479 (m), 1459 (m), 1438 (m), 1152 (w), 1082 (w), 1037 (w), 948 (m), 870 (w), 805 (w), 763 (s), 747 (s), 682 (m), 632 (w), 597 (w), 565 (w), 535 (m), 480 (m), 459 (m), 406 (w)

References

(1) Finikova, O. S.; Aleshchenkov, S. E.; Briñas, R. P.; Cheprakov, A. V; Carroll, P. J.; Vinogradov, S. A. Synthesis of Symmetrical Tetraaryltetranaphtho[2,3]Porphyrins. *J. Org. Chem.* **2005**, *70* (12), 4617–4628. https://doi.org/10.1021/jo047741t.

(2) Bae, Y. J.; Christensen, J. A.; Kang, G.; Zhou, J.; Young, R. M.; Wu, Y.-L.; Van Duyne, R. P.; Schatz, G. C.; Wasielewski, M. R. Substituent Effects on Energetics and Crystal Morphology Modulate Singlet Fission in 9,10-Bis(Phenylethynyl)Anthracenes. *J. Chem. Phys.* **2019**, *151* (4), 44501. https://doi.org/10.1063/1.5110411.

(3) Lin, Y.-S. et al., Long-Range Corrected Hybrid Density Functionals with Improved Dispersion Corrections *J. Chem. Theory Comput.* **2013**, *9* (1), 263–272.

(4) Weigend F. and Ahlrichs R., Balanced basis sets of split valence, triple zeta valence and quadruple zeta valence quality for H to Rn: Design and assessment of accuracy, *Phys. Chem. Chem. Phys.,* **2005**,*7*, 3297.

(5) Weigend F., Accurate Coulomb-fitting basis sets for H to Rn, *Phys. Chem. Chem. Phys.*, **2006**, *8*, 1057.

(6) Neese, F.; Wennmohs, F.; Becker, U.; Riplinger, C. The ORCA quantum chemistry program package, *J. Chem. Phys.*, **2020**, *152*, 224108.

(7) Grimme S., Antony J., Ehrlich S. and Krieg H., A consistent and accurate *ab initio* parametrization of density functional dispersion correction (DFT-D) for the 94 elements H-Pu *J.Chem.Phys.*, **2010**, *132*, 154104.

(8) Kruse H., Grimme S., A geometrical correction for the inter- and intra-molecular basis set superposition error in Hartree-Fock and density functional theory calculations for large systems *J.Chem.Phys*., **2012**, *136*, 154101.

(9) Ásgeirsson V. et al., Nudged Elastic Band Method for Molecular Reactions Using Energy-Weighted Springs Combined with Eigenvector Following. *J Chem Theory Comput* **2021**, *17* (8), 4929-4945.

(10) I. Fdez. Galv´an, M. Vacher, A. Alavi, C. Angeli, F. Aquilante, J. Autschbach, J. J. Bao, S. I. Bokarev, N. A. Bogdanov, R. K. Carlson, L. F. Chibotaru, J. Creutzberg, N. Dattani, M. G. Delcey, S. S. Dong, A. Dreuw, L. Freitag, L. M. Frutos, L. Gagliardi, F. Gendron, A. Giussani, L. Gonz´alez, G. Grell, M. Guo, C. E. Hoyer, M. Johansson, S. Keller, S. Knecht, G. Kovaˇcevi´c, E. K¨allman, G. Li Manni, M. Lundberg, Y. Ma, S. Mai, J. a. P. Malhado, P. r. Malmqvist, P. Marquetand, S. A. Mewes, J. Norell, M. Olivucci, M. Oppel, Q. M. Phung, K. Pierloot, F. Plasser, M. Reiher, A. M. Sand, I. Schapiro, P. Sharma, C. J. Stein, L. K. Sørensen, D. G. Truhlar, M. Ugandi, L. Ungur, A. Valentini, S. Vancoillie, V. Veryazov, O. Weser, T. A. Wesolowski, P.-O. Widmark, S. Wouters, A. Zech, J. P. Zobel, R. Lindh, *Journal of Chemical Theory and Computation* **2019**, *15*, 5925–5964.

(11) Siewertsen, R. et al. Superior Z → E and E → Z Photoswitching Dynamics of Dihydrodibenzodiazocine, a Bridged Azobenzene, by S1(Nπ*) Excitation at λ = 387 and

490 Nm. *Phys. Chem. Chem. Phys.* **2011**, *13* (3), 1054–1063.
(12) Dunning T. H., Gaussian basis sets for use in correlated molecular calculations. I. The atoms boron through neon and hydrogen *The Journal of Chemical Physics* **1989**, *90*, 1007–1023.
(13) Hehre W. J., Stewart R. F., People J. A., Self-Consistent Molecular-Orbital Methods. I. Use of Gaussian Expansions of Slater-Type Atomic Orbitals *The Journal of Chemical Physics* **1969**, *51*, 2657–2664.
(14) Becke, A. D. Density-functional thermochemistry. III. The role of exact exchange *The Journal of Chemical Physics* **1993**, *98*, 5648–5652.
(15) Weigend, F. A fully direct RI-HF algorithm: Implementation, optimised auxiliary basis sets, demonstration of accuracy and efficiency *Phys. Chem. Chem. Phys.* **2002**, *4*, 4285–4291.
(16) Caldeweyher E., Ehlert S., Hansen A., Neugebauer H., Spicher S., Bannwarth C., Grimme S., A generally applicable atomic-charge dependent London dispersion correction *The Journal of Chemical Physics* **2019**, *150*, 154122.
(17) TURBOMOLE V7.4 2019, a development of University of Karlsruhe and Forschungszentrum Karlsruhe GmbH, 1989-2007, TURBOMOLE GmbH, since 2007; available from http://www.turbomole.com
(18) Moormann, W.; Langbehn, D.; Herges, R., *Synthesis* **2017**, *49*, 3471-3475.
